# Supplementary material for: Mitochondrial Hsp60/10 Client Protein Decline Reveals Braak/Tau- and Cognition-Linked Proteostasis Vulnerabilities in Alzheimer’s Disease
Source: bioRxiv. 2026 Jun 30:2026.06.25.734545. Preprint. [Version 1] doi: 10.64898/2026.06.25.734545 (PMC13345081; doi:10.64898/2026.06.25.734545)
Supplement: Supplement 2 [file NIHPP2026.06.25.734545v1-supplement-2.pdf]

bioRxiv preprint doi: https://doi.org/10.64898/2026.06.25.734545; this version posted June 30, 2026. The copyright holder for this preprint (which was not certified by peer review) is the author/funder, who has granted bioRxiv a license to display the preprint in perpetuity. It is made available under a CC-BY 4.0 International license.

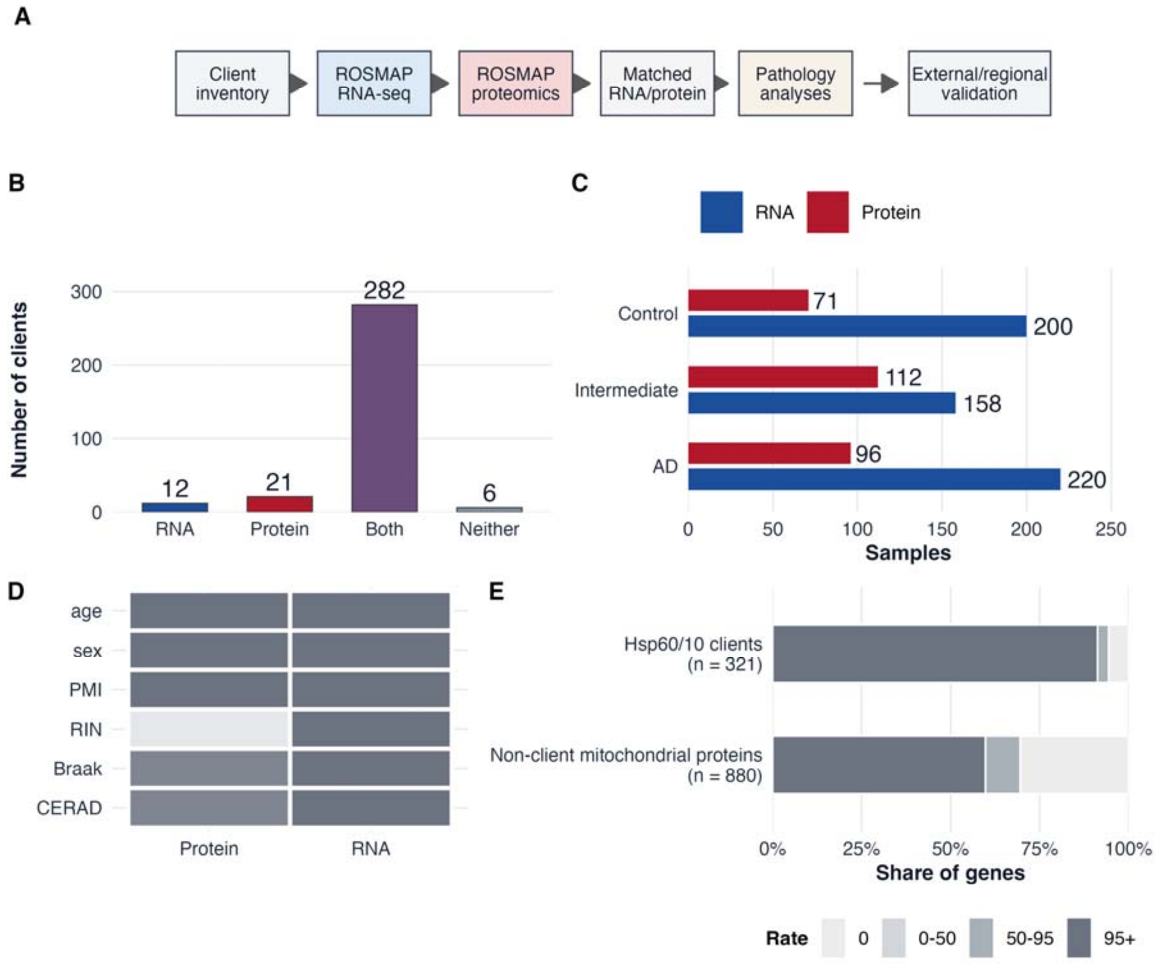

Supplemental Figure 1. Cohort, detection, and Hsp60/10 client-set definition.

Summary of the analytical workflow, Hsp60/10 client detection across ROSMAP RNA-seq and TMT proteomics, diagnostic-stage sample composition, covariate availability, and protein detection-rate categories. (A) Analysis workflow integrating a published Hsp60/10 client inventory with ROSMAP RNA-seq, TMT proteomics, matched RNA/protein subsets, pathology analyses, and external or regional validation analyses. (B) Detection of the 321-client reference inventory across RNA-seq and proteomics: RNA only (n = 12 clients), protein only (n = 21), both RNA and protein (n = 282), and neither (n = 6). (C) RNA-seq and TMT proteomics sample counts across NCI, MCI, and AD diagnostic-stage groups: RNA n = 200, 158, and 220 and

bioRxiv preprint doi: https://doi.org/10.64898/2026.06.25.734545; this version posted June 30, 2026. The copyright holder for this preprint (which was not certified by peer review) is the author/funder, who has granted bioRxiv a license to display the preprint in perpetuity. It is made available under a CC-BY 4.0 International license.

protein n = 71, 112, and 96 for NCI, MCI, and AD respectively. (D) Availability of covariates and pathology variables used across RNA and protein analyses. (E) Protein detection-rate categories for Hsp60/10 clients (n = 321) and non-client mitochondrial proteins (n = 880). Detection-rate bins were 0%, >0–50%, 50–95%, and ≥95%.

bioRxiv preprint doi: https://doi.org/10.64898/2026.06.25.734545; this version posted June 30, 2026. The copyright holder for this preprint (which was not certified by peer review) is the author/funder, who has granted bioRxiv a license to display the preprint in perpetuity. It is made available under a CC-BY 4.0 International license.

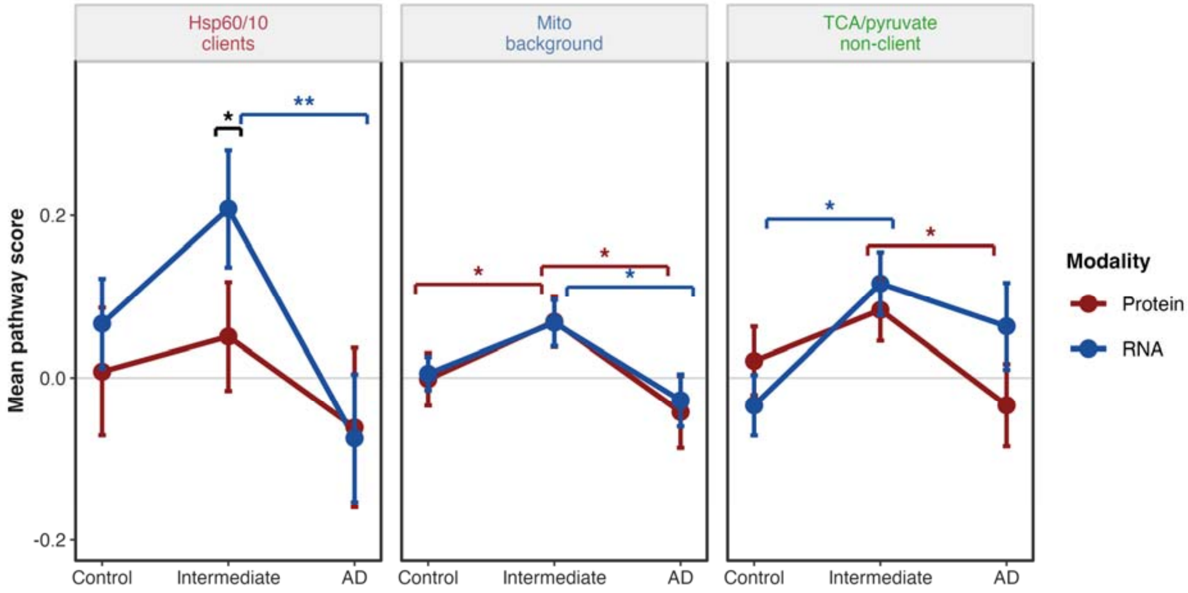

Supplemental Figure 2. Matched-individual RNA/protein sensitivity analysis.

Matched ROSMAP samples with both RNA-seq and TMT proteomics were used to test whether stage-associated remodeling patterns were preserved when RNA and protein were compared within the same individuals. Mean standardized pathway scores are shown across NCI, MCI, and AD groups for the detected Hsp60/10 client network, broad mitochondrial background, and TCA/pyruvate non-Hsp60/10 comparator pathway. Red indicates protein and blue indicates RNA. Points show group means; error bars show SEM. Colored brackets indicate within-modality stage comparisons. No significant protein-versus-RNA differences were detected within the same stage. *P < 0.05, **P < 0.01, ***P < 0.001.

bioRxiv preprint doi: https://doi.org/10.64898/2026.06.25.734545; this version posted June 30, 2026. The copyright holder for this preprint (which was not certified by peer review) is the author/funder, who has granted bioRxiv a license to display the preprint in perpetuity. It is made available under a CC-BY 4.0 International license.

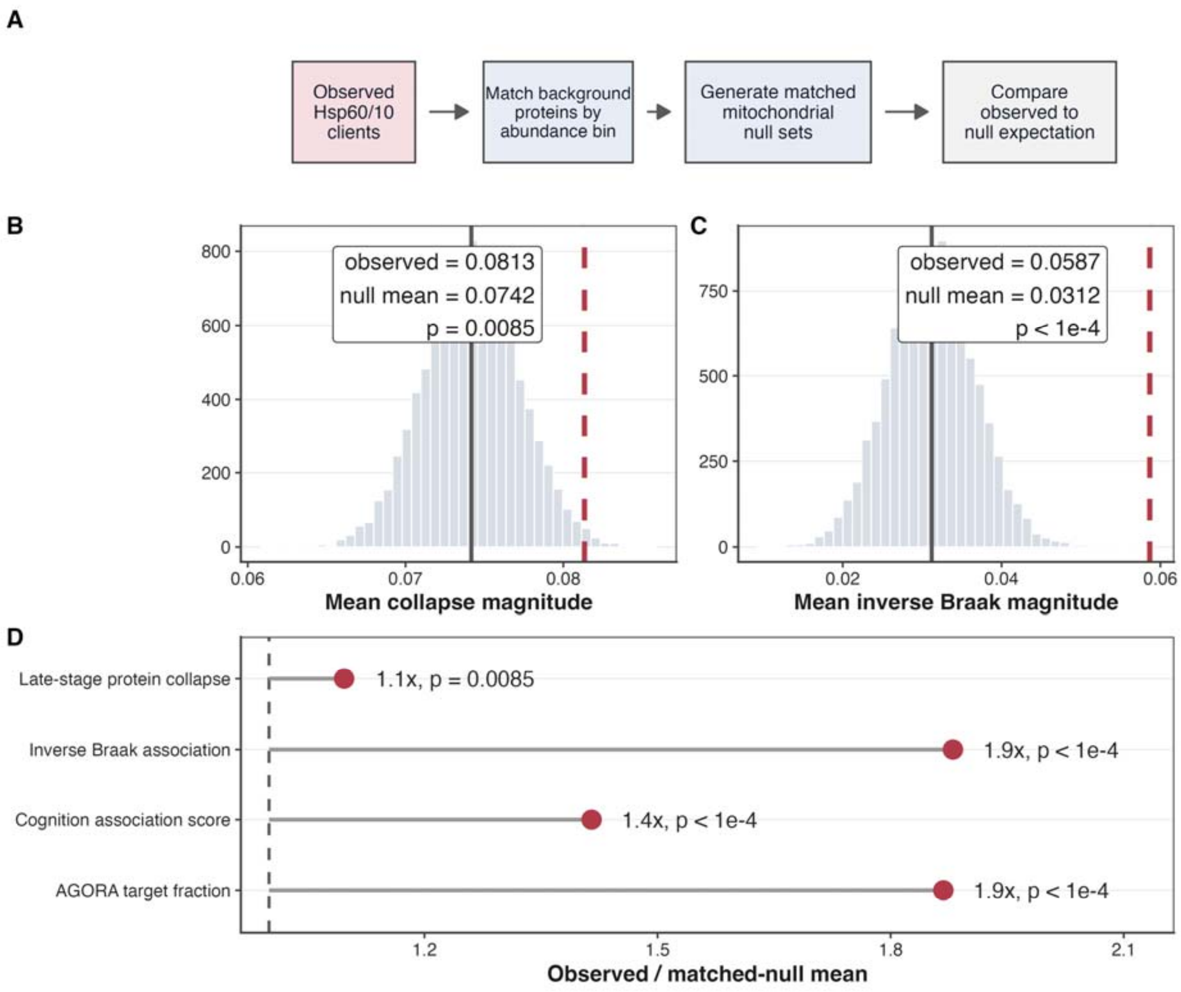

Supplemental Figure 3. Specificity against non-client mitochondrial proteins.

Observed Hsp60/10 client metrics were compared with 10,000 abundance-matched non-client mitochondrial null sets to test whether client-network signals exceeded matched mitochondrial background expectations. Values greater than 1 indicate stronger observed Hsp60/10 signal than matched-null expectation. The observed set contained n = 306 detected Hsp60/10 clients, and the matched pool contained n = 609 non-client mitochondrial proteins. (A) Schematic of the abundance-matched null strategy. (B) Null distribution of mean late-stage protein collapse magnitude: observed = 0.0813, null mean = 0.0742, empirical P = 0.0085. The dashed red line marks the observed Hsp60/10 client mean; the gray line marks the null mean. (C) Null

bioRxiv preprint doi: https://doi.org/10.64898/2026.06.25.734545; this version posted June 30, 2026. The copyright holder for this preprint (which was not certified by peer review) is the author/funder, who has granted bioRxiv a license to display the preprint in perpetuity. It is made available under a CC-BY 4.0 International license.

distribution of mean inverse Braak association magnitude: observed = 0.0587, null mean = 0.0312, empirical $P < 1.0 \times 10^{-4}$, with values bound by 10,000 null iterations. (D) Observed-to-null ratios were 1.1 for late-stage protein collapse, 1.9 for inverse Braak association, 1.4 for cognition association score, and 1.9 for Agora target fraction; empirical P = 0.0085 for collapse and $P < 1.0 \times 10^{-4}$, with values bound by 10,000 null iterations, for the remaining metrics.

bioRxiv preprint doi: https://doi.org/10.64898/2026.06.25.734545; this version posted June 30, 2026. The copyright holder for this preprint (which was not certified by peer review) is the author/funder, who has granted bioRxiv a license to display the preprint in perpetuity. It is made available under a CC-BY 4.0 International license.

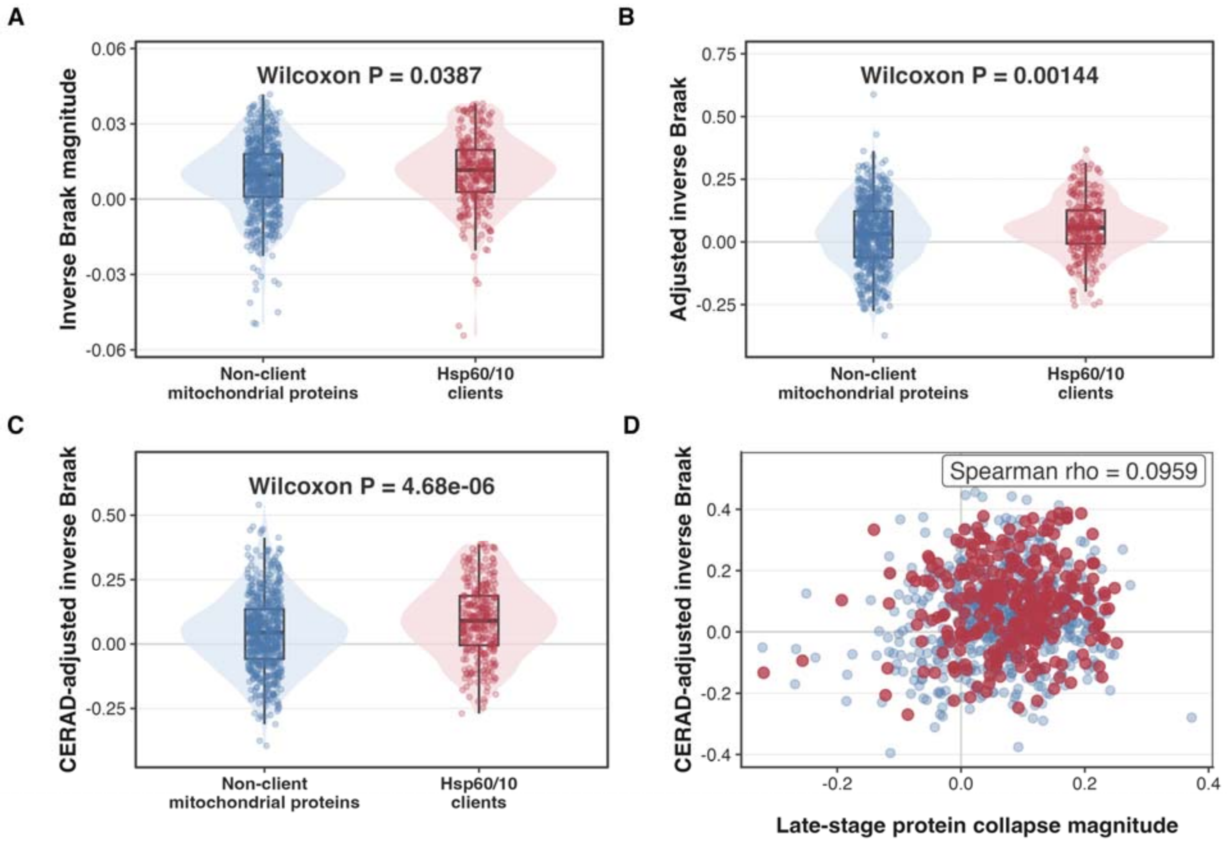

Supplemental Figure 4. Pathology model robustness of Hsp60/10 client vulnerability.

Alternative pathology models were used to test whether Hsp60/10 client vulnerability was robust to model specification. Positive inverse Braak values indicate lower protein abundance with higher Braak pathology. Hsp60/10 clients (n = 306 proteins) were compared with detected non-client mitochondrial proteins (n = 609 proteins) using two-sided Wilcoxon rank-sum tests. (A) Unadjusted inverse Braak magnitude from Spearman associations, P = 0.0387. (B) Covariate-adjusted inverse Braak beta values from models including Braak stage, age, sex, and PMI, P = 0.00144. (C) CERAD-adjusted inverse Braak beta values from models including Braak stage, CERAD score, age, sex, and PMI, P = 4.68 x 10^{-6}. (D) Late-stage collapse magnitude was

bioRxiv preprint doi: https://doi.org/10.64898/2026.06.25.734545; this version posted June 30, 2026. The copyright holder for this preprint (which was not certified by peer review) is the author/funder, who has granted bioRxiv a license to display the preprint in perpetuity. It is made available under a CC-BY 4.0 International license.

correlated with CERAD-adjusted inverse Braak beta across mitochondrial proteins using Spearman correlation, rho = 0.0959.

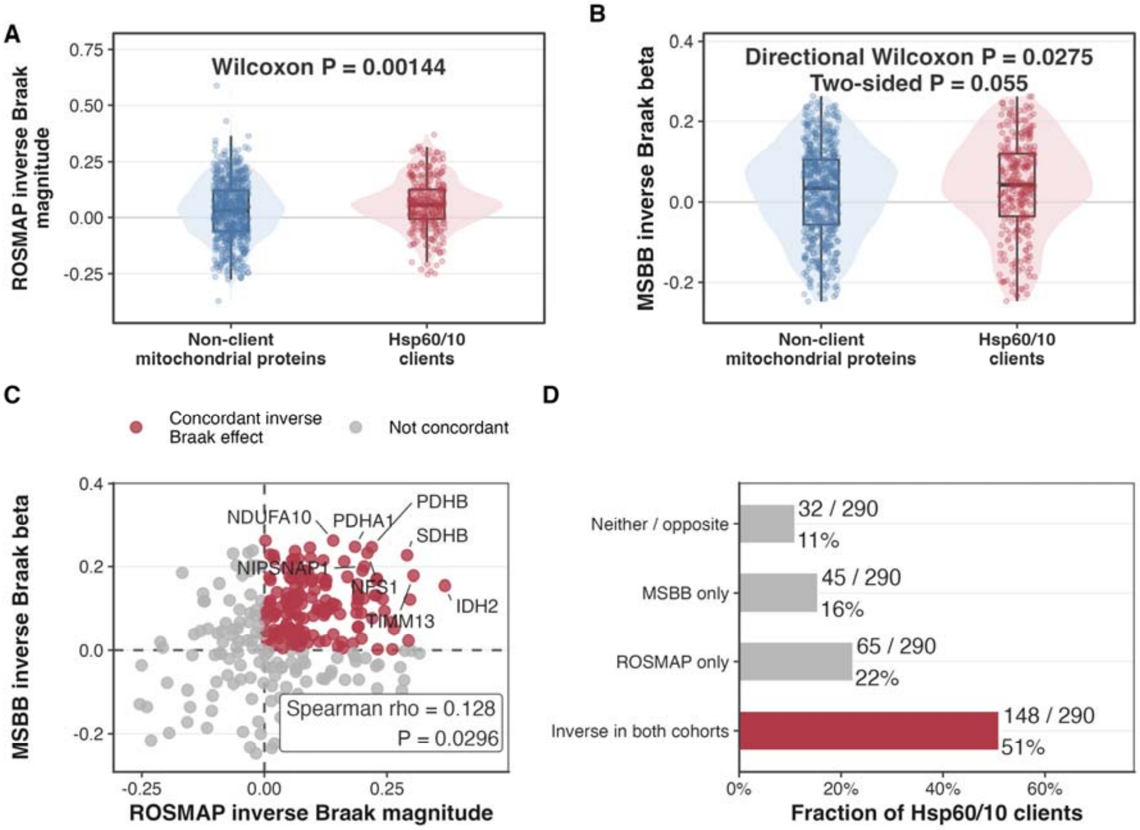

Supplemental Figure 5. External MSBB cross-cohort validation.

bioRxiv preprint doi: https://doi.org/10.64898/2026.06.25.734545; this version posted June 30, 2026. The copyright holder for this preprint (which was not certified by peer review) is the author/funder, who has granted bioRxiv a license to display the preprint in perpetuity. It is made available under a CC-BY 4.0 International license.

Independent MSBB proteomics data were used to evaluate whether Hsp60/10 client pathology coupling observed in ROSMAP was directionally reproduced across cohorts. Positive inverse Braak values indicate lower protein abundance with greater pathology burden. (A) ROSMAP inverse Braak magnitude compared between Hsp60/10 clients (n = 306 proteins) and non-client mitochondrial proteins (n = 609 proteins) using a two-sided Wilcoxon rank-sum test, P = 0.00144. (B) MSBB inverse Braak beta values compared between Hsp60/10 clients and non-client mitochondrial proteins using a two-sided Wilcoxon rank-sum test, one-sided P = 0.0275; two-sided P = 0.055. (C) Gene-level comparison of ROSMAP and MSBB inverse Braak effects for Hsp60/10 clients detected in both cohorts (n = 290 proteins) using Spearman correlation, rho = 0.128, P = 0.0296. Red points indicate concordant inverse Braak effects in both cohorts; gray points indicate non-concordant effects. Dashed lines mark zero effect. (D) Concordance categories among the n = 290 shared clients are shown as counts and percentages.

bioRxiv preprint doi: https://doi.org/10.64898/2026.06.25.734545; this version posted June 30, 2026. The copyright holder for this preprint (which was not certified by peer review) is the author/funder, who has granted bioRxiv a license to display the preprint in perpetuity. It is made available under a CC-BY 4.0 International license.

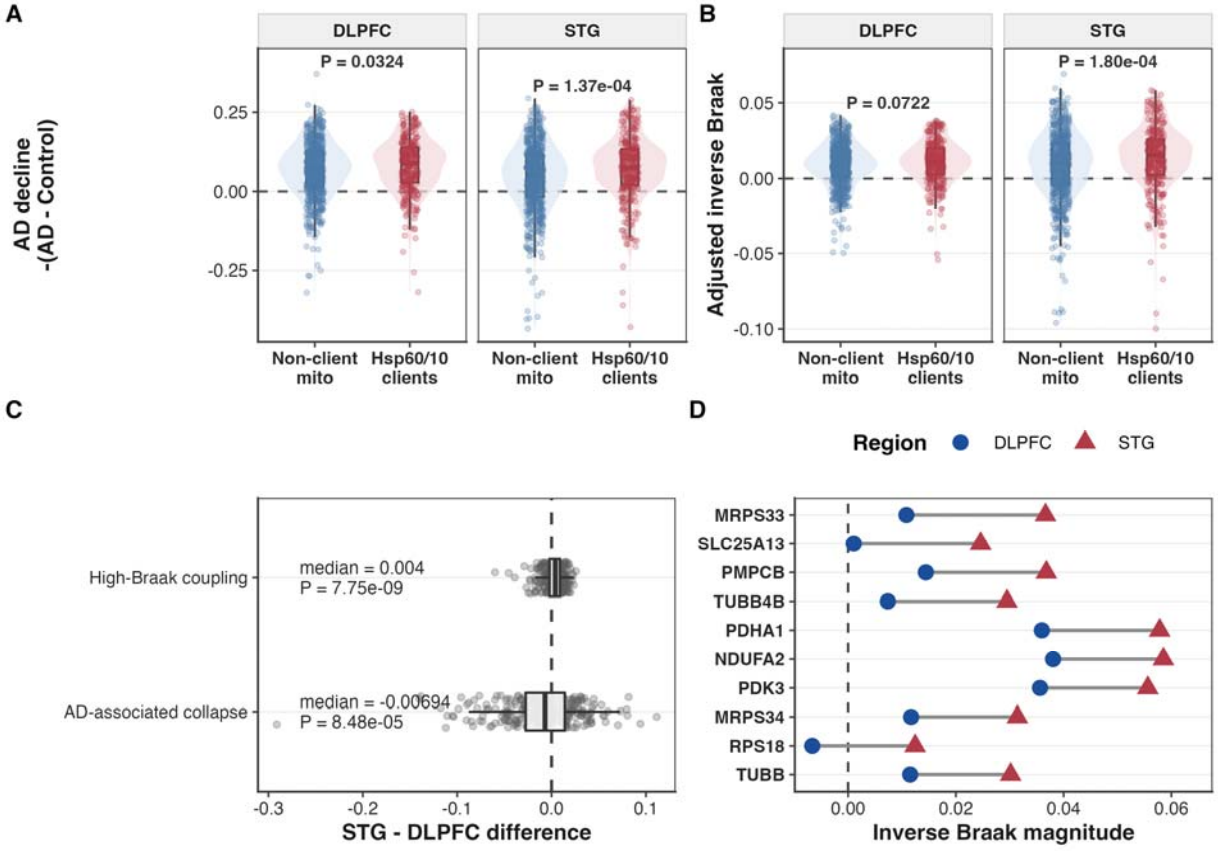

Supplemental Figure 6. Regional proteomics validation of Hsp60/10 client vulnerability.

Regional proteomics analyses in dorsolateral prefrontal cortex (DLPFC) and superior temporal gyrus (STG) tested whether Hsp60/10 clients showed regional evidence of AD- and pathology-associated protein vulnerability. Hsp60/10 clients were compared with detected non-client mitochondrial proteins. (A) AD-associated protein decline in DLPFC and STG, oriented so positive values indicate greater decline in AD versus NCI: DLPFC P = 0.0324; STG P = 1.37×10^{-4}. (B) Adjusted inverse Braak-associated protein decline: DLPFC P = 0.0722, STG P = 1.80×10^{-4}. (C) STG-minus-DLPFC differences among Hsp60/10 clients were tested using paired Wilcoxon signed-rank tests: high-Braak coupling median = 0.004, P = 7.75×10^{-9}; AD-associated collapse median = -0.00694, P = 8.48×10^{-5}. Positive values indicate stronger signals in STG; negative values indicate stronger signals in DLPFC. (D) Hsp60/10 clients ranked by

bioRxiv preprint doi: https://doi.org/10.64898/2026.06.25.734545; this version posted June 30, 2026. The copyright holder for this preprint (which was not certified by peer review) is the author/funder, who has granted bioRxiv a license to display the preprint in perpetuity. It is made available under a CC-BY 4.0 International license.

STG-minus-DLPFC adjusted inverse Braak magnitude. Points show descriptive, region-specific values, and connecting lines show within-gene regional shifts.
